# Does *Trypanosoma cruzi* (Chagas, 1909) (Kinetoplastida: Trypanosomatidae) modify the antennal phenotype of *Triatoma dimidiata* (Latreille, 1811) (Hemiptera: Triatominae)?

**DOI:** 10.1186/s13071-022-05587-y

**Published:** 2022-12-14

**Authors:** Irving J. May-Concha, Maryrose J. Escalante-Talavera, Jean-Pierre Dujardin, Etienne Waleckx

**Affiliations:** 1 Facultad de Medicina Veterinaria Y Zootecnia Campus II, Universidad Autónoma de Chiapas, Carretera Emiliano Zapata Km. 8, 29060 Tuxtla Gutierrez, CHIS México; 2 Laboratorio de Parasitología, Centro de Investigaciones Regionales “Dr Hideyo Noguchi”, Universidad Autónoma de Yucatán, Mérida, México; 3 Institut de Recherche pour le Développement, UMR INTERTRYP IRD, CIRAD, Université de Montpellier, Montpellier, France

**Keywords:** Triatomines, Chagas disease, Host manipulation, Phenotypic plasticity

## Abstract

**Background:**

*Triatoma dimidiata* is a vector of the protozoan parasite *Trypanosoma cruzi*, the etiologic agent of Chagas disease. Phenotypic plasticity allows an organism to adjust its phenotype in response to stimuli or environmental conditions. Understanding the effect of *T. cruzi* on the phenotypic plasticity of its vectors, known as triatomines, has attracted great interest because of the implications of the parasite–triatomine interactions in the eco-epidemiology and transmission of the etiologic agent of Chagas disease. We investigated if the infection of the vector with *T. cruzi* may be associated with a change in the antennal phenotype of sylvatic, domestic, and laboratory-reared populations of *T. dimidiata*.

**Methods:**

The abundance of each type of sensillum (bristles, basiconic, thick- and thin-walled trichoid) on the antennae of *T.* *cruzi*-infected and non-infected *T.* *dimidiata* reared in the laboratory or collected in sylvatic and domestic ecotopes were measured under light microscopy and compared using Kruskal–Wallis non-parametric tests and permutational multivariate analysis of variance.

**Results:**

We found significant differences between sensilla patterns of infected and non-infected insects within sylvatic and domestic populations. Conversely, we found no significant differences between sensilla patterns of infected and non-infected insects within the laboratory-reared population. Besides, for sylvatic and domestic populations, sexual dimorphism tended to be increased in infected insects.

**Conclusion:**

The differences observed in infected insects could be linked to higher efficiency in the perception of odor molecules related to the search for distant mates and hosts and the flight dispersal in search of new habitats. In addition, these insects could have a positive effect on population dynamics and the transmission of *T.* *cruzi*.

**Graphical Abstract:**

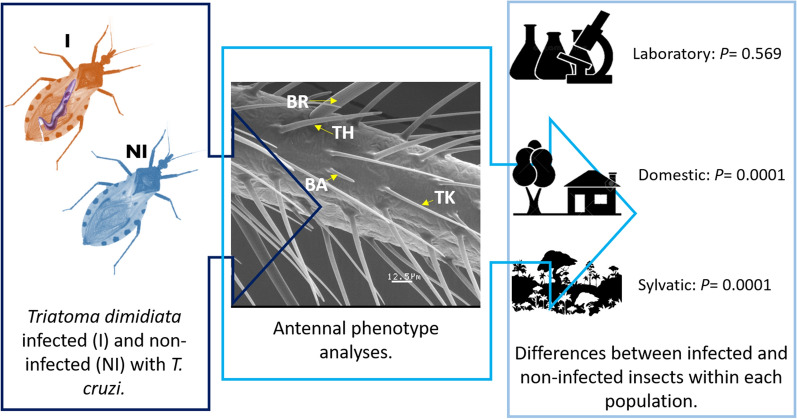

**Supplementary Information:**

The online version contains supplementary material available at 10.1186/s13071-022-05587-y.

## Background

Phenotypic plasticity is of great interest in ecology and evolution because it allows an organism to actively adjust its phenotype in response to stimuli or environmental conditions [1–8]. The response may or may not be adaptive, and it may involve changes in morphology, physiological state, behavior, or some combination of these [9]. Besides, phenotypic plasticity is also widely recognized as an important factor for the evolution, population biology, and ecological interactions of many species [10–13]; thus, it is a major mechanism of ecological adaptation [14]. Most information on phenotypic plasticity comes mainly from social insects [14–16], triatomines [17–20], grasshoppers [21, 22], and butterflies [13, 23].

Triatomine nymphs and adults of both sexes are strict blood-sucking insects that feed on vertebrate species available in their habitat [20]. Their olfactory system plays an important role in many behavioral contexts, such as host-seeking, refuges and mate finding, alarm and aggregation behaviors, as well as avoidance of natural enemies [24]. In triatomines, the antennal phenotype (AP) comprises the type and number of sensilla (classified as mechanoreceptors and chemoreceptors) distributed on the antennae. Sensilla act as an interface between the external and internal environments of insects (inter and intraspecific communication), capturing different stimuli from the external environment and directing them to the central nervous system [24–26]. This then triggers specific behavioral responses, such as the selection of a host for feeding, oviposition behavior, mate finding, and alarm and aggregation behaviors [27–33].

AP has been widely used as a sensitive marker to distinguish populations of triatomines [33–35]. In certain species or complexes, AP analysis complements other phenotypic and genetic characteristics [34, 36–39] or provides evidence for species differentiation [40, 41]. On the other hand, previous studies have established that the antennal sensilla of triatomines may show a degree of morphological variability between populations that seem to be associated with adaptations based on the sensorial requirements of different habitats [17, 37]. The number of sensilla may also vary because of selection pressure, sex, infection by a microorganism, and feeding habits [37, 39, 42–46]. Such changes show the degree of phenotypic plasticity exhibited by the species [17]. Importantly, different studies have shown an absence of a correlation between the number of chemoreceptors and the total antenna length, length of antenna segments, and the number of each type of sensillum arranged over them [47, 48].

As vectors of the parasite *Trypanosoma* *cruzi* (Chagas, 1909) (Kinetoplastida: Trypanosomatidae), the causal agent of Chagas disease, the insects of the subfamily Triatominae (Hemiptera: Reduviidae), have special relevance in Latin America [49]. The parasite is transmitted to humans and other animals when feces or urine of infected insects come into contact with mucous membranes or damaged areas of mammal skin [50]. The co-evolution between triatomines and *T.* *cruzi* has promoted the development of powerful and sophisticated strategies, which can modify a wide range of physiological processes of the insects, including those related to the input, development, and discharge of the parasite [51]. The existence of these modifications as a characteristic of an association between *T.* *cruzi* and triatomines could be the consequence of different adaptive or nonadaptive scenarios (e.g., adaptive host manipulation) [52, 53]. While several works have analyzed the mechanisms associated with *T. cruzi*–vector dynamics (e.g., biotic and abiotic factors) to understand the *T. cruzi*–triatomine interactions, under a co-evolutionary scenario [54], literature about how the parasites may influence the insects is more limited, and the studies have mainly been focused on the parasite’s effects on four patterns of the vector behavior: life-history traits, feeding, defecation, and dispersion/locomotion [55]. Different studies have found negative effects of *T. cruzi* infection on vector survival [56–59], fecundity [59, 60], post-embryonic development [59, 61, 62], behavior [55, 63–68], and physiological processes [55, 60, 69–71], while other studies have not identified these effects on patterns of alimentation/defecation [56, 72, 73], development, and reproduction [74–76]. Overall, most of these studies determined that the effects of *T. cruzi* are species-dependent, age-dependent, sex-dependent, and even environment/physiology-dependent.

Although the AP, effects of *T. cruzi,* and phenotypic plasticity of the triatomines have been extensively studied [17, 34, 54], the phenotypic plasticity linked to the infection with *T. cruzi* in triatomines has not been investigated so far. In this study, we evaluated the changes in the AP of *Triatoma dimidiata* (Latreille, 1811) according to *T. cruzi* infection. More specifically, we investigated whether *T. cruzi* infection was associated with AP and sexual dimorphism modifications.

## Methods

### Insects

Laboratory-reared *T. dimidiata* came from a colony maintained for the past 10 years at the Parasitology Laboratory of the Regional Research Center Dr. Hideyo Noguchi, Autonomous University of Yucatan. New insects have been periodically added to this colony to avoid inbreeding depression. The insects were reared and maintained for 11 generations under controlled conditions (27 ± 1 °C, 70 ± 5% RH, a photoperiod of 12:12 [L:D] h), and were fed on immobilized pigeons [*Columba livia* Gmelin, 1789 (Aves, Columbidae)]. The domestic and sylvatic populations were composed of insects collected during entomological surveillance in 2018 inside and outside human dwellings of the rural village of Teya (25° 02′ 55″ N, 89° 04′ 25″ W), Yucatan, Mexico. The closest human dwellings were 3.5 km from the sylvatic site. The study was approved by the Institutional Bioethics Committee of the Autonomous University of Yucatan.

### *Trypanosoma cruzi*

For infection of triatomines, the “V strain,” a TcI strain of *T. cruzi* originally isolated from a *T. dimidiata* specimen and maintained in the laboratory by cyclical passages in BALB/c adult mice, was used.

### Infection of the laboratory-reared triatomines with *T. cruzi*

After a 2-week starvation period, the initial infection of the laboratory-reared triatomines was carried out with nymphs that had just molted to their fifth instar. Nymphs were fed ad libitum on BALB/c mice 15 days after they were infected with 1 × 10^6^ parasites ml^−1^ of blood (i.e., during the parasite's exponential stage of growth [65]). Approximately 30 days after infection, we corroborated the infection status through examination of a fecal drop observed under a light microscope at ×40 magnification. Control group insects were fed under the same conditions on non-infected mice. The nymphs of both groups were maintained under rearing conditions and were fed fortnightly on infected/non-infected mice until they molted to the adult stage.

### Assessment of the infection of domestic and sylvatic populations with *T. cruzi*

For *T. dimidiata* collected in natural conditions (i.e., domestic and sylvatic populations), we evaluated the infection with *T. cruzi* by amplifying parasite DNA from each bug midgut by polymerase chain reaction (PCR), using TCZ primers as described previously [77]. Based on the results obtained by PCR, each population had a group of infected and non-infected insects.

### Antennal preparation

We examined a total of 130 antennae of *T. cruzi*-infected and non-infected females and males from the sylvatic, domestic, and laboratory-reared populations of *T. dimidiata* (Table 1). One right antenna per specimen was removed using fine forceps and scissors. Antennae were processed with sodium hydroxide 4% for 6 h at 60 °C and then neutralized with glacial acetic acid 5% for 2 min. This procedure allowed cuticle diaphanization and enabled the identification and counting of the sensilla using a Zeiss Primostar^®^ stereo microscope at ×400 magnification. The number and type of sensilla on antennal segments was counted manually using a procedure reported in previous works [33]. The ventral side of the three distal segments of the antennae (P: pedicel, F1: flagellum 1, and F2: flagellum 2) was evaluated by identifying and counting sensilla including bristles (BR), thin-walled trichoid (TH), thick-walled trichoid (TK), and basiconic (BA) (nomenclature according to Catalá and Schofield [36]), thus giving a total of 12 morphological variables. The person who performed the measurements was unaware of the bug’s infection status to avoid any bias (MJET).

**Table 1:** Number of *T. dimidiata* specimens used in this study. Population, sex and infection status of the specimens are indicated

Populations	Infected		Non-infected		Overall
	♀	♂	♀	♂	
Laboratory-reared	10	10	10	11	41
Domestic	10	10	10	10	40
Sylvatic	10	10	13	16	49
Overall	30	30	33	37	130

♀: female and ♂: male

### Data analysis

Differences in the AP between *T. cruzi*-infected (I) and non-infected (NI) insects were explored in the overall population, within each sex, within each population (i.e., sylvatic, domestic, and laboratory-reared), and within each sex within each population using univariate and multivariate analyses. Means and standard deviations of abundance were calculated for each type of sensilla (chemoreceptors: BR, TH, TK, and mechanoreceptors: BA) and antennal segment (pedicel, flagellum 1, and flagellum 2). As original data and their transformations were not normally distributed using Shapiro–Wilk tests [78], Kruskal–Wallis non-parametric tests were used for univariate analyses, followed by pairwise comparisons [79]. Data were analyzed with the Minitab Statistical Software, version 17 (Minitab, Inc., PA, USA). In all cases, *P* < 0.05 was considered statistically significant. Moreover, the sources of variation of the AP were assessed using two-way permutational multivariate analysis of variance (PERMANOVA) on Bray–Curtis similarity matrices of square root with 9999 permutations. These analyses were conducted in PAST version 3.05.

## Results

### Overall data

Abundances of the sensilla found for all the *T. dimidiata* specimens included in this study are shown in Additional file 1: Table S1. All the insects’ antennae presented three types of chemoreceptors (TH, TK, and BA) and one mechanoreceptor (BR) on the three segments. The average number of sensilla per insect was 669.52 ± 176.45. Overall, the TH sensillum of the pedicel (P-TH) was the most abundant (183.42 ± 92.70), while the BR sensillum of the flagellum 2 (F2-BR) was the least abundant (17.45 ± 12.51). The pedicel was the segment with the highest number of sensilla (322.42 ± 115.54) while the flagellum 2 was the segment with the lowest number of sensilla (149.63 ± 54.43).

### AP of *T. cruzi*-infected and non-infected *T. dimidiata*

Differences in each sensillum on the three antennal segments between infected and non-infected insects in the overall population, within each sex, within each population (i.e., sylvatic, domestic, and laboratory-reared), and within each sex within each population are summarized in Table 2.

**Table 2:** Comparisons of the abundance of each sensillum between infected and non-infected insects overall population, within each sex, within each population, and within each sex within each population of *Triatoma dimidiata*

Factor	Pedicel				Flagellum 1				Flagellum 2			
	BR	BA	TH	TK	BR	BA	TH	TK	BR	BA	TH	TK
Overall population (I vs. NI)	–	**	–	–	–	–	–	*	–	–	–	–
Within females (I F vs. NI F)	–	–	*	–	–	–	–	–	–	–	–	–
Within males (I M vs. NI M)	–	–	–	–	–	–	–	**	–	–	–	–
Within domestic insects (I D vs. NI D)	*	–	*	*	***	*	–	*	*	***	*	–
Within sylvatic insects (I S vs. NI S)	–	***	–	–	***	**	–	***	**	**	*	**
Within laboratory-reared insects (I L vs. NI L)	–	–	–	–	–	–	–	–	–	–	–	–
Within females of the domestic population (F D I vs. F D NI)	–	–	*	–	–	–	–	–	–	*	*	–
Within females of the sylvatic population (F S I vs. F S NI)	–	***	–	–	**	**	–	–	*	*	–	*
Within females of the laboratory-reared population (F L I vs. F L NI)	–	–	–	–	–	–	–	–	–	–	–	–
Within males of the domestic population (M D I vs. M D NI)	*	–	–	–	***	–	–	–	*	***	–	–
Within males of the sylvatic population (M S I vs. M S NI)	–	*	–	–	**	–	–	***	*	–	*	–
Within males of the laboratory-reared population (M L I vs. M L NI)	–	–	–	–	–	–	–	–	–	–	–	–

*BR* bristles, *BA* basiconic, *TH* thin-walled trichoid, *TK* thick-walled trichoid, *F* female and *M* male, *I* infected, *NI* non-infected, *D* domestic, *S* sylvatic, and *L* laboratory-reared. Asterisks represent a significant difference between infected and non-infected insects (*P* < 0.05*; *P* < 0.01**; *P* < 0.001***; – no difference)

#### Overall population

When infected and non-infected insects were compared, significantly more BA sensilla on pedicel (P-BA) and TK sensilla on flagellum 1 (F1-TK) were observed in infected insects (Kruskal–Wallis test, *P* = 0.007 and *P* = 0.01, respectively).

#### Within each sex

When infected and non-infected insects were compared for each sex (I females vs, NI females; I males vs, NI males), significantly fewer TH sensilla on pedicel (P-TH) were observed in infected females (Kruskal–Wallis test, *P* = 0.04). Conversely, significantly more TK sensilla on flagellum 1 (F1-TK) were observed in infected males (Kruskal–Wallis test, *P* = 0.008).

#### Within each population

In the domestic population, when infected and non-infected insects were compared, significantly more BR sensilla on pedicel (P-BR) were observed in infected insects (Kruskal–Wallis test, *P* = 0.01). On the other hand, significantly fewer TH and TK sensilla on pedicel, BR, BA, TK sensilla on flagellum 1, and BR, BA, TH sensilla on flagellum 2 were observed in infected insects (Kruskal–Wallis test, *P* < 0.05 in all cases). Additionally, the two-way PERMANOVA test associated the infection with *T. cruzi* with the AP of the domestic population (*F* = 7.15; *P* = 0.0001), while the sex and the interaction infection*sex did not have a significant association with the AP (*F* = 1.51; *P* = 0.177 and *F* = 1.188; *P* = 0.299, respectively; Table 3).

**Table 3:** Two-way PERMANOVA based on the Bray–Curtis distance matrix assessing the sources of variation of the antennal phenotype of *T. dimidiata* populations

Source of variation	Sum of squares	Mean square	*F*-test	*P*-value
Domestic population				
Infection	0.188	0.188	7.151	0.0001
Sex	0.039	0.0.39	1.5179	0.1776
Interaction	0.031	0.031	1.1887	0.2993
Sylvatic population				
Infection	0.209	0.209	7.418	0.0001
Sex	0.121	0.121	4.288	0.0021
Interaction	0.103	0.013	0.368	0.125
Laboratory-reared				
Infection	0.031	0.031	0.708	0.569
Sex	0.083	0.083	1.869	0.104
Interaction	0.052	0.052	0.126	0.473

*P*-values are based on 9999 permutations

In the sylvatic population, when infected and non-infected insects were compared, significantly more BA sensilla on pedicel, BR, BA, and TK sensilla on flagellum 1, and BR, BA, TH and TK sensilla on flagellum 2 were observed in infected insects (Kruskal–Wallis test, *P* < 0.05 in all cases). The two-way PERMANOVA test associated the infection with *T. cruzi* and the sex with the AP of the sylvatic population (*F* = 7.41; *P* = 0.0001 and *F* = 4.28; *P* = 0.002, respectively), while the interaction infection*sex did not have a significant association with the AP (*F* = 0.368; *P* = 0.125; Table 3).

Finally, in the laboratory-reared population, when infected and non-infected insects were compared, no difference in the number of sensilla was observed (Kruskal–Wallis test, *P* > 0.05 in all cases). In the same way, the two-way PERMANOVA test did not reveal significant association of the infection with *T. cruzi*, of the sex and of the interaction infection*sex with the AP of laboratory-reared insects (*P* > 0.05; Table 3).

#### Within each sex within each population

Differences in the abundance of each sensillum on the three antennal segments between infected and non-infected insects within each sex within each population are shown in Additional file 1: Table S1 and are summarized in Table 2.

##### Domestic population

When infected and non-infected females of the domestic population were compared, significantly fewer TH sensilla on pedicel and flagellum 2, and BA sensilla on flagellum 2 (Kruskal–Wallis, *P* < 0.05 in all cases) were observed. On the other hand, when infected and non-infected males of the domestic population were compared, significantly more BR sensilla on pedicel (P-BR) (Kruskal–Wallis test, *P* = 0.01) were observed. Moreover, when infected and non-infected males of the domestic population were compared, significantly fewer BR sensilla on flagellum 1 and flagellum 2, and BA sensilla on flagellum 2 (Kruskal–Wallis, *P* < 0.05 in all cases) were observed.

##### Sylvatic population

When infected and non-infected females of the sylvatic population were compared, significantly more BA sensilla on the three segments of the antennae, BR sensilla on flagellum 1 and flagellum 2, and TK sensilla on flagellum 2 (Kruskal–Wallis test, *P* < 0.05 in all cases) were observed. On the other hand, when infected and non-infected males of the sylvatic population were compared, significantly more BA sensilla on pedicel, BR and TK sensilla on flagellum 1, and BR and TH sensilla on flagellum 2 (Kruskal–Wallis test, *P* < 0.05 in all cases) were observed.

##### Laboratory-reared population

In the laboratory-reared population, there were no differences in the abundance of each sensillum between infected and non-infected females and between infected and non-infected males (Kruskal–Wallis test, *P* > 0.05).

### Sexual dimorphism of *T. cruzi*-infected and non-infected insects

Differences in the abundances of each sensillum between non-infected females and males, and between infected females and males in the overall population, and within each population, are summarized in Table 4.

**Table 4:** Comparisons of the abundances of each sensillum between infected females and males and between non-infected females and males overall population, and within each population of *Triatoma dimidiata*

Factor	Pedicel				Flagellum 1				Flagellum 2			
	BR	BA	TH	TK	BR	BA	TH	TK	BR	BA	TH	TK
Overall non-infected insects (NI F vs, NI M)	–	–	–	–	–	–	–	–	–	–	–	–
Overall infected insects (I F vs, I M)	–	–	**	–	–	–	–	–	–	–	–	–
Within non-infected domestic insects (F D NI vs, M D NI)	–	–	–	–	–	–	–	–	–	–	–	–
Within infected domestic insects (F D I vs, M D I)	–	–	–	–	–	–	*	–	–	–	–	–
Within non-infected sylvatic insects (F S NI vs, M S NI)	–	–	*	–	–	–	**	–	–	–	–	–
Within infected sylvatic insects (F S I vs, M S I)	–	**	*	–	–	*	–	–	–	–	–	–
Within non-infected laboratory-reared insects (F L NI vs, M L NI)	–	–	–	–	–	–	–	–	–	–	–	–
Within infected laboratory-reared insects (F L I vs, M L I)	–	–	–	–	–	–	–	–	–	–	–	–

*BR* bristles, *BA* basiconic, *TH* thin-walled trichoid, *TK* thick-walled trichoid, *F* female, *M* male, *I* infected, *NI* non-infected, *D* domestic, *S* sylvatic, *L* laboratory-reared. Asterisks represent a significant difference between infected and non-infected insects (*P* < 0.05*, *P* < 0.01**, *P* < 0.001***, – no difference)

#### Overall population

When non-infected females and males were compared, no significant difference in the abundance of each sensillum was observed (Kruskal–Wallis test, *P* > 0.05). However, when infected females and males were compared, significantly more TH sensilla on pedicel (P-TH) were observed in males (Kruskal–Wallis test, *P* = 0.002).

#### Domestic population

When non-infected females and males were compared, no significant difference in the abundance of each sensillum was observed (Kruskal–Wallis test, *P* > 0.05). However, when infected females and males were compared, significantly more TH sensilla on flagellum 1 (F1-TH) were observed in males (Kruskal–Wallis test, *P* = 0.01).

#### Sylvatic population

When non-infected females and males were compared, significantly more TH sensilla on pedicel (P-TH) and flagellum 1 (F1-TH) were observed in males (Kruskal–Wallis test, *P* = 0.02 and *P* = 0.003, respectively).

When infected females and males were compared, a significant difference in the abundance of TH sensilla on pedicel (P-TH) was still observed (Kruskal–Wallis test, *P* = 0.04), while the difference in the abundance of TH sensilla on flagellum 1 (F1-TH) was not observed anymore. However, significantly more BA sensilla on pedicel (P-BA) and flagellum 1 (F1-BA) were observed in females (Kruskal–Wallis test, *P* = 0.003 and *P* = 0.04, respectively).

#### Laboratory-reared population

In the laboratory-reared population, there was no sexual dimorphism in infected and non-infected insects (Kruskal–Wallis test, *P* > 0.05).

## Discussion

Phenotypic plasticity has been analyzed in different triatomine species in response to ecological factors [17, 35, 80], or to assess the effect of ecotope [18], food source [19], environment [35, 43, 81, 82], and sex [80]. The present study is the first to analyze phenotypic plasticity related to the infection with *T. cruzi* in domestic, sylvatic, and laboratory-reared populations of *T. dimidiata*.

Our results show that there is an association between the infection status and the AP of *T. dimidiata*, at least in natural conditions. Indeed, we observed that infected and non-infected insects from the domestic and sylvatic populations showed significant differences in the abundance of some sensilla types. Besides, our results show that the sexual dimorphism tends to increase in *T. cruzi*-infected natural populations. Nevertheless, these differences in the abundance of some sensilla types between infected and non-infected insects, and the increased sexual dimorphism in infected insects was not observed in the laboratory-reared population. Consequently, while we were unable to evidence in this study a causal relationship between the infection with *T. cruzi* and the observed AP differences between infected and non-infected insects in natural populations of *T. dimidiata*, we could not exclude it either because the laboratory-reared insects were infected during their fifth development stage. If a causal relationship between *T. cruzi* infection and AP exists, this suggests that we should have established the infection in the earliest development stages to observe this effect, since insects infected in early development stages are more likely to be manipulated, as Poulin et al. [83] have suggested. However, more laboratory research is needed to understand how long it would take for *T. cruzi* to modify the AP of *T. dimidiata* and if there is a relation between AP changes and the parasite load in these vectors [84].

Therefore, the question remains whether the observed morphological differences are explained by the direct effect of *T. cruzi* infection and host manipulation, are evolutionary responses to selection at the population level, or are the consequences of different causal factors such as microecological influences [85].

Determining why and how host manipulation by parasites evolves is a fascinating but challenging question for evolutionary biologists. Pioneer authors addressing this question [86, 87] proposed that host changes probably occurred after the establishment of complex life cycles involving more than one host species. Ramirez-Gonzalez et al. [66] determined the effect of *T. cruzi* on the motor activity of fifth-stage nymphs of *Triatoma* *longipennis* Usinger, 1939 and *Triatoma phyllosoma* (Burmeister, 1835) infected during the second-stage nymph. On the other hand, Depickere et al. [55] determined the effect of *T. cruzi* on the aggregation behavior of *Triatoma infestans* (Klug, 1834) captured in the field and naturally infected. Recent studies with *T. infestans* have shown that infected insects after 45 days present changes in their circadian locomotor activity and feeding and defecation patterns [68, 73].

In our study, infected insects of the domestic and sylvatic populations showed, in general, significantly more BR sensilla compared with non-infected insects. These mechanoreceptors are associated with habitat selection rather than host selection [36], with the perception of mechanical stimuli related to the microhabitat [88]. This suggests that infected insects could have greater capacities for adaptation or colonization of new habitats as compared with non-infected insects.

On the other hand, these mechanoreceptors also allow insects to perceive vibratory signals (through stridulation) during mating, and variations in the air current [88–91]. Moreover, they play an important role in the orientation toward odor-laden currents [92]. Various studies have determined that the infection by *T. cruzi*, can impair the fecundity, fertility, and mating performance of triatomines (e.g., Fellet et al. [60]). An increase in these mechanoreceptors suggests that infected insects may benefit from copulation frequency and searching mating pairs, although reproductive success could be affected because of the infection. However, several functional aspects of these mechanoreceptors are unknown, and for this reason, further studies aimed at analyzing these contexts are needed to gain a better understanding of the functionality associated with habitat selection and the search for mating pairs.

Concerning the chemoreceptors (BA, TH and TK), it has previously been reported that BA sensilla have an olfactory and/or gustative function for the detection of habitats, shelters, hosts, mating pairs and related to the perception of sex-pheromone [93–95]. Besides, these sensilla seem involved in the detection of presumed pheromones in conspecific feces [96, 97]. The multiparous TH sensilla were first described in *T. infestans* by Bernard [93]. The function of these sensilla may be associated with reproductive activities [37]. They respond to a range of fatty acids—particularly pyruvic and lactic—and amyl acetate, and to breathing [93, 98]. On the other hand, although TK sensilla have been shown to predominate in triatomines [34], their chemosensory function has not been confirmed [93, 99]. However, Bernard [93] suggested that they may respond only to special compounds such as pheromones, thus acting as olfactory sensilla [100], as has been shown in the insect *Cimex lectularius* (Linnaeus, 1758) (Hemiptera: Cimicidae) [101]. In our study, variation in the olfactory sensitivity associated with *T. cruzi* infection in the domestic and sylvatic populations is suggested. Indeed, in these populations, infected and non-infected insects showed significant differences in the abundance of some specific chemoreceptors. In the sylvatic population, the infection with *T. cruzi* was associated with more abundance of chemoreceptors. In a natural context, triatomines rely heavily on their sense of smell to locate, detect, and orientate toward a host from which they feed [34]. The evidence indicates infected *T. longipennis* and *Triatoma pallidipennis* react more quickly to human odor than non-infected [66]. Infected *Mepraia spinolai* (Porter, 1934) orient toward their host twice as fast, and their number of bites is duplicated [64]. Some of these behavioral changes could promote vector competence, which encompasses the ability to acquire, maintain, and transmit a pathogen [68]. Assuming the hypothesis of sensory modulation by the parasite [71, 92], it is possible that the infected insects of the sylvatic population generate an increase in the antennal receptors because of the wide range of hosts in the sylvatic ecotope and to enhance parasite transmission probability [77, 102, 103]. Moreover, this increase may enhance the capacity for dispersal and invasion of different habitats [47, 81], and efficiency in the perception of odor molecules in the search of distant mates and hosts and for flight dispersal in search of new habitats, as it has been suggested by other authors [82, 102, 104–106], thus conferring an advantage to *T. cruzi*.

Nevertheless, infected insects of the domestic populations showed a decrease in some chemoreceptors. The occurrence of these modifications as characteristic of a parasite–host system may be the consequence of an adaptive process (i.e., adaptive host manipulation by the parasite or compensatory response by the host). Also, it may be an after-effect of the presence of the parasite that allows insects to reduce their investment in costly or reduced-use structures [52, 107].

Several studies have provided information about the sexual dimorphism in non-infected triatomines from different species, populations, rearing, and ecotopes [38, 80, 88]. However, our study reports for the first time the sexual dimorphism in the AP of infected insects of *T. dimidiata*. In general, the sexual dimorphism observed in infected insects of *T. dimidiata* was based on an increase in the abundance of TH sensilla in infected males and/or an increase of BA sensilla in infected females. These chemoreceptors have an olfactory function for the detection of sexual pairs, habitats, and hosts as mentioned above. Evidence of this study and previous works [48, 81, 108] suggest that the sexual dimorphism in the AP may be linked to the perception of molecules related to sexual behavior and to differences in sensing sexual pheromones, as has been suggested by other authors (e.g., May-Concha et al. [34]; Souza et al. [82]). May-Concha [109] provided information on a chemical signal produced during *T. dimidiata* mating, since fewer mating attempts were observed when the opening of female glands was occluded. Besides, that study describes a chemical signal which promotes the attraction of males to volatiles emitted by females and to mating couples [30]. On the other hand, based on previous works on olfactory receptors [24–26], we can propose that the increased abundance of TH chemo-sensilla in infected males contributes to a greater efficiency in the perception of odor molecules involved in sexual communication compared with infected females. In contrast, we can hypothesize that the increased abundance of BA chemo-sensilla in infected females contributes to a greater efficiency in the perception of host odors compared with infected males. Therefore, the increase in the odor perception in infected insects may elicit a positive effect on vector population dynamics and could enhance the vectorial transmission of *T. cruzi*. Future studies should examine in-depth the effect of the parasite on other aspects of the behavior of triatomine insects, such as aggregation, alarm, feeding, excretion/defecation, and host foraging, which could constitute epidemiologically relevant behavioral changes, and evaluate sexual behavioral changes in adults, which could impact the growth of triatomine populations.

## Conclusion

To our knowledge, this is the first work that relates an association of the AP with the *T. cruzi* infection status of *T. dimidiata*. Although we could not demonstrate any causal relationship, we revealed a clear association between the natural infection status of *T. dimidiata* and its antennal phenotypic variation. The differences observed in infected insects could be linked to higher efficiency in the olfactory perception related to the search for distant mates and hosts and the flight dispersal in search of new habitats. In addition, these insects could have a positive effect on population dynamics and the transmission of *T. cruzi*.

## Supplementary Information

**Additional file 1: Table S1. **Abundances of each sensillum on the three antennal segments in infected and non-infected insects of each sex within each population of *T.* *dimidiata*. The data shown are the means and standard deviation. N=130. TH: thin-walled trichoid; TK: thick-walled trichoid; BA: basiconic; BR: bristles. The number between parentheses represents the number of specimens analyzed. The number between clasps represents the standard deviation of the data. I = infected, NI = non-infected, D = domestic, L = laboratory-reared, S = sylvatic, F = female, M = male. Different letters indicate significant differences between infected and non-infected insects of the same sex within each population (Kruskal–Wallis tests; P < 0.05).

## Data Availability

The datasets used and/or analyzed during the present study are available from the corresponding author upon reasonable request.

## Abbreviations

AP: Antennal phenotype
P: Pedicel
F1: Flagellum 1
F2: Flagellum 2
BR: Bristles
TH: Thin-walled trichoid
TK: Thick-walled trichoid
BA: Basiconic
I: *T.* *cruzi*-Infected
NI: *T.* *cruzi*-Non-infected
