# Supplementary material for: Does Trypanosoma cruzi (Chagas, 1909) (Kinetoplastida: Trypanosomatidae) modify the antennal phenotype of Triatoma dimidiata (Latreille, 1811) (Hemiptera: Triatominae)?
Source: Parasit Vectors. 2022 Dec 14;15:466. doi: 10.1186/s13071-022-05587-y (PMC9749310; doi:10.1186/s13071-022-05587-y)
Supplement: Supplementary file 1 — Additional file 1: Table S1. Abundances of each sensillum on the three antennal segments in infected and non-infected insects of each sex within each population of T. dimidiata. The data shown are the means and standard deviation. N=130. TH: thin-walled trichoid; TK: thick-walled trichoid; BA: basiconic; BR: bristles. The number between parentheses represents the number of specimens analyzed. The number between clasps represents the standard deviation of the data. I = infected, NI = non-infected, D = domestic, L = laboratory-reared, S = sylvatic, F = female, M = male. Different letters indicate significant differences between infected and non-infected insects of the same sex within each population (Kruskal–Wallis tests; P < 0.05). [file 13071_2022_5587_MOESM1_ESM.docx]

**Additional data**

**Table S1.** Abundances of each sensillum on the three antennal segments in infected and non-infected insects of each sex within each population of *T. dimidiata*. The data shown are the means and standard deviation. N=130. TH: thin-walled trichoid; TK: thick-walled trichoid; BA: basiconic; BR: bristles. The number between parentheses represents the number of specimens analyzed. The number between clasps represents the standard deviation of the data. I = infected, NI = non-infected, D = domestic, L = laboratory-reared, S = sylvatic, F = female, M = male. Different letters indicate significant differences between infected and non-infected insects of the same sex within each population (Kruskal-Wallis tests; P < 0.05).

| **Infection** | **Population** | **Sex** | **Pedicel** | | | |  |  | **Flagellum 1** | | | |  |  | **Flagellum 2** | | | |  | **Total antennae** |
| --- | --- | --- | --- | --- | --- | --- | --- | --- | --- | --- | --- | --- | --- | --- | --- | --- | --- | --- | --- | --- |
|  |  |  | **TH** | **TK** | **BA** | **BR** | **∑** |  | **TH** | **TK** | **BA** | **BR** | **∑** |  | **TH** | **TK** | **BA** | **BR** | **∑** |  |
| I | D | F(10) | 119.80 [46.72]^a^ | 61.40 [31.85] | 18.20 [10.98] | 40.20 [10.13] | **239.60**  **[71.26]** |  | 47.70 [18.57] | 74.20 [13.30] | 21.00 [15.03] | 36.80 [28.34] | **179.70**  **[58.22]** |  | 22.80 [12.90]^a^ | 78.40 [12.40] | 25.70 [15.43]^a^ | 15.20 [7.73] | **142.10**  **[31.94]** | **561.40**  **[136.27]** |
| NI | D | F(10) | 207.30 [92.11]^b^ | 80.30 [45.55] | 12.60 [5.54] | 38.10 [11.12] | **338.30**  **[118.08]** |  | 54.20 [17.94] | 107.10 [42.56] | 26.50 [9.98] | 53.60 [24.36] | **241.40**  **[47.23]** |  | 39.20 [15.31]^b^ | 87.50 [30.47] | 47.00 [19.20]^b^ | 23.80 [14.47] | **197.50**  **[47.49]** | **777.20**  **[117.04]** |
| I | D | M(10) | 181.10 [84.27] | 52.30 [17.96] | 17.80 [8.32] | 47.50 [11.47]^a^ | **298.70**  **[94.85]** |  | 66.00 [17.56] | 71.90 [7.87] | 19.60 [6.00] | 19.10 [4.28]^a^ | **176.60**  **[21.61]** |  | 26.10 [6.97] | 68.90 [14.58] | 18.50 [5.52]^a^ | 12.00 [3.02]^a^ | **125.50**  **[23.24]** | **600.80**  **[104.57]** |
| NI | D | M(10) | 206.60 [95.54] | 89.20 [52.36] | 19.20 [7.44] | 33.20 [10.61]^b^ | **348.20**  **[112.33]** |  | 54.70 [22.06] | 76.20 [12.67] | 28.40 [10.47] | 64.10 [28.26]^b^ | **223.40**  **[28.42]** |  | 27.60 [10.11] | 73.90 [16.00] | 41.10 [14.78]^b^ | 15.90 [3.41]^b^ | **158.50**  **[27.30]** | **730.10**  **[124.74]** |
| I | L | F(10) | 120.60 [64.10] | 71.50 [57.90] | 15.80 [9.44] | 45.60 [17.28] | **253.50**  **[131.75]** |  | 52.60 [22.14] | 107.60 [39.89] | 24.80 [12.39] | 19.00 [7.72] | **204.00**  **[62.29]** |  | 20.40 [10.11] | 74.80 [31.73] | 19.70 [11.72] | 12.30 [5.40] | **127.20**  **[52.39]** | **584.70**  **[230.18]** |
| NI | L | F(10) | 128.50 [62.87] | 92.50 [55.11] | 20.10 [10.48] | 57.80 [16.40] | **298.90**  **[101.27]** |  | 51.30 [32.77] | 88.80 [37.29] | 29.00 [11.85] | 23.40 [10.52] | **192.50**  **[30.46]** |  | 31.30 [29.75] | 78.30 [27.07] | 27.00 [8.86] | 17.70 [10.10] | **154.30**  **[23.74]** | **645.70**  **[117.87]** |
| I | L | M(10) | 195.80 [108.20] | 52.50 [21.44] | 17.80 [6.89] | 53.50 [17.06] | **319.60**  **[121.81]** |  | 54.90 [19.87] | 98.00 [33.37] | 23.90 [12.50] | 21.60 [9.18] | **198.40**  **[53.05]** |  | 20.30 [8.04] | 78.90 [27.49] | 18.40 [7.81] | 13.50 [4.03] | **131.10**  **[39.70]** | **649.10**  **[184.65]** |
| NI | L | M(11) | 184.55 [152.11] | 43.45 [23.01] | 15.18 [9.56] | 47.45 [11.86] | **290.63**  **[179.79]** |  | 53.18 [25.53] | 73.55 [23.62] | 20.36 [10.39] | 21.09 [10.82] | **168.18**  **[49.11]** |  | 27.64 [19.10] | 68.27 [17.40] | 21.00 [10.32] | 21.82 [14.70] | **138.72**  **[49.31]** | **597.54**  **[212.06]** |
| I | S | F(10) | 159.60 [64.44] | 75.80 [32.19] | 54.40 [23.32]^a^ | 64.20 [11.38] | **354.00**  **[70.89]** |  | 61.80 [13.71] | 99.00 [50.50] | 44.20 [20.27]^a^ | 50.30 [26.02]^a^ | **255.30**  **[82.57]** |  | 24.50 [7.65] | 118.00 [54.42]^a^ | 37.40 [19.88]^a^ | 39.20 [22.78]^a^ | **219.10**  **[92.90]** | **828.40**  **[227.79]** |
| NI | S | F(13) | 181.69 [91.02] | 66.38 [38.29] | 15.92 [4.46]^b^ | 57.77 [13.08] | **321.76**  **[134.63]** |  | 50.08 [18.02] | 65.00 [22.32] | 19.77 [8.64]^b^ | 17.77 [7.10]^b^ | **152.61**  **[45.05]** |  | 21.08 [11.86] | 75.77 [34.35]^b^ | 17.54 [8.69]^b^ | 11.85 [4.74]^b^ | **126.23**  **[42.81]** | **600.61**  **[186.17]** |
| I | S | M(10) | 222.60 [49.69] | 74.70 [36.37] | 24.60 [10.80]^a^ | 54.80 [19.10] | **376.70**  **[25.98]** |  | 67.10 [24.12] | 93.80 [17.80]^a^ | 27.20 [10.67] | 29.10 [9.56]^a^ | **217.20**  **[46.86]** |  | 38.20 [22.48]^a^ | 92.60 [29.92] | 25.30 [11.52] | 20.40 [13.33]^a^ | **176.50**  **[65.34]** | **770.40**  **[106.86]** |
| NI | S | M(16) | 252.06 [72.57] | 62.69 [29.90] | 14.94 [4.42]^b^ | 61.44 [11.67] | **391.12**  **[89.32]** |  | 74.81 [23.32] | 68.50 [17.20]^b^ | 22.50 [7.28] | 18.75 [6.43]^b^ | **184.56**  **[33.62]** |  | 21.69 [10.25]^b^ | 71.25 [25.49] | 19.00 [8.43] | 10.94 [4.02]^b^ | **122.87**  **[37.09]** | **689.56**  **[131.26]** |
| **Average** |  | **(130)** | **183.42**  **[92.70]** | **68.05**  **[39.39]** | **20.14**  **[14.16]** | **50.81**  **[16.05]** | **322.42**  **[115.54]** |  | **57.97**  **[22.56]** | **83.97**  **[31.45]** | **25.28**  **[12.75]** | **30.25**  **[21.87]** | **197.47**  **[54.77]** |  | **26.38**  **[15.59]** | **79.92**  **[30.44]** | **25.88**  **[15.12]** | **17.45**  **[12.51]** | **149.63**  **[54.43]** | **669.52**  **[176.45]** |
